# Comparative Genomics of Mycoplasma: Analysis of Conserved Essential Genes and Diversity of the Pan-Genome

**DOI:** 10.1371/journal.pone.0035698

**Published:** 2012-04-20

**Authors:** Wei Liu, Liurong Fang, Mao Li, Sha Li, Shaohua Guo, Rui Luo, Zhixin Feng, Bin Li, Zhemin Zhou, Guoqing Shao, Huanchun Chen, Shaobo Xiao

**Affiliations:** 1 Division of Animal Infectious Diseases, State Key Laboratory of Agricultural Microbiology, College of Veterinary Medicine, Huazhong Agricultural University, Wuhan, People's Republic of China; 2 Institute of Veterinary Medicine, Jiangsu Academy of Agricultural Sciences, Nanjing, People's Republic of China; 3 Environmental Research Institute, University College Cork, Cork, Ireland; Institut de Genetique et Microbiologie, France

## Abstract

*Mycoplasma*, the smallest self-replicating organism with a minimal metabolism and little genomic redundancy, is expected to be a close approximation to the minimal set of genes needed to sustain bacterial life. This study employs comparative evolutionary analysis of twenty *Mycoplasma* genomes to gain an improved understanding of essential genes. By analyzing the core genome of mycoplasmas, we finally revealed the conserved essential genes set for mycoplasma survival. Further analysis showed that the core genome set has many characteristics in common with experimentally identified essential genes. Several key genes, which are related to DNA replication and repair and can be disrupted in transposon mutagenesis studies, may be critical for bacteria survival especially over long period natural selection. Phylogenomic reconstructions based on 3,355 homologous groups allowed robust estimation of phylogenetic relatedness among mycoplasma strains. To obtain deeper insight into the relative roles of molecular evolution in pathogen adaptation to their hosts, we also analyzed the positive selection pressures on particular sites and lineages. There appears to be an approximate correlation between the divergence of species and the level of positive selection detected in corresponding lineages.

## Introduction

Mycoplasmas are widespread in nature as parasites of humans, mammals, reptiles, fish, arthropods, and plants [1]. As a conditional pathogenic organism, it associates with various diseases, including pneumonia, arthritis, meningitis and chronic urogenital tract disease [2]. Although they are the smallest self-replicating organisms, both commensal forms and pathogenic forms are diverse. With a minimal metabolism and little genomic redundancy, the genome of *Mycoplasma* is expected to be a close approximation to the minimal set of genes needed to sustain bacterial life [3]. An early projection proposed a minimal gene set composed of 206 genes based on the analysis of eight free-living and endosymbiotic bacterial genomes [4]. More recently, Glass *et al.* [5] performed a global transposon mutagenesis study and identified 100 putatively nonessential genes in *M. genitalium*. Logically, the remaining 387 genes presumably constitute the set of essential genes. However, these data greatly exceed theoretical projections of how many genes comprise a minimal genome, as proposed by Gil *et al.* [4].

Natural selection leads to the fixation of essential genes and can delete nonessential genes in a wide range of species [6], [7]. This process is similar, but more robust than manual mutagenesis studies. Through long term evolution form a more conventional progenitor in the *Firmicutes* taxon [8], *Mycoplasmas* have undergone a process of massive genome reduction [1]. These wall-less bacteria are obligate parasites that live in relatively unchanging niches requiring little adaptive capability. *M. genitalium*, a human urogenital pathogen, is the extreme manifestation of this genomic parsimony, having only 482 protein-coding genes and the smallest genome of any known free-living organism capable of being grown in axenic culture [9]. Thus, with little genomic redundancy and contingencies for different environmental conditions, *Mycoplasmas* are regarded as optimal microbes to perform genes essentiality studies [5]. Along with the burgeoning increase in *Mycoplasma* genome sequence data, this would appear to be the right time to explore gene essentiality from a comparative genomics perspective.

An enormous genetic diversity exists in the mycoplasmas, yet how much diversity is functional, and what are the important adaptations that serve to partition species into different niches? *M. hyopneumoniae* and *M. hyorhinis* are the causal agents of swine mycoplasmosis. The former causes a mild, chronic pneumonia of swine and results in deactivation of mucociliary functions [10]. This agent is infective for a single host species, but the mechanisms of host specificity are unknown. The latter is responsible for respiratory tract and arthritis disease in swine [1]. *M. hyorhinis* is generally considered a swine pathogen, yet is most commonly infect laboratory cell lines, implying that it can thrive among different species of cell lines [11]. A strong link between *M. hyorhinis* and human cancer was reported recently by Huang *et al.* [12], who used a monoclonal antibody against the unique *M. hyorhinis*–specific protein p37 to detect mycoplasma in over 600 carcinoma tissues from a variety of organs. The study indicated that up to 56% of gastric carcinoma and 55% of colon carcinoma biopsies were positive for *M. hyorhinis* [12]. With a similar genome size, *M. hyorhinis* and *M. hyopneumoniae* exhibit high levels of functional diversity. Interest has therefore shifted to questions of why *M. hyorhinis* can thrive among different species of cell lines.

This paper communicates the results of three major analyses. In the first analysis, we present the details of a comparative analysis of twenty *Mycoplasma* strains and investigate the conserved essential genes set for mycoplasma survival. For the second analysis, phylogenomic reconstructions based on 3,355 homologous groups allows robust estimation of phylogenetic relatedness among mycoplasma strains. The third analysis employs the branch-site method to assess positive selection pressures on particular sites and lineages. There appears to be an approximate correlation between the divergence of species and the level of positive selection detected in corresponding lineages.

## Results

### Diversity of *Mycoplasmataceae* family: core genome *vs.* flexible gene pool

**Figure 1:**
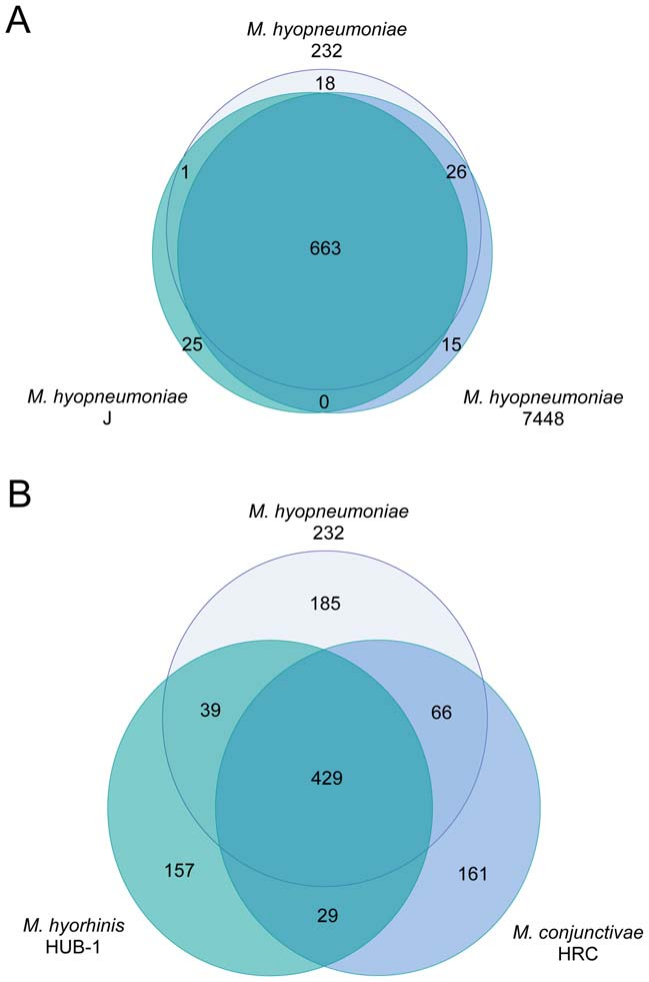
Venn diagram for two sets of three taxa. Above are taxa of the same species and below are taxa of different species. The surfaces are approximately proportional to the number of genes.

The number of protein coding genes per genome within the various strains and species of mycoplasmas is relatively similar (ranging from 475 to 1,037; Table 1), but the gene composition of these genomes is much more variable. Based on the gene content table (obtained as described in Materials and Methods; Table S1), three *M. hyopneumoniae* strains share about 95% of their genes, and three different species of mycoplasmas share only around 71% of their genes (Figure 1). This latter result appears to be independent of the particular strains or species involved in the comparison. Even with the inclusion of 20 genomes, the pan-genome size of *Mycoplasmas* appears not to be determined, and we estimate that the size probably surpasses 8,000 genes. This huge pan-genome size may be a reflection of their different lifestyles in distinct ecological niches. Within species, the pan-genome size also remains uncertain, although our estimates suggest that the pan-genome size of *M. hyopneumoniae* is smaller.

**Table 1:** Bacterial Strains Used in This Study.

Mollicute strains	Host	CDS	Genome size (bp)	Accession	Citation
***U. urealyticum*** ** serovar 10 str. ATCC 33699**	Human	646	874478	CP001184	-
***U. parvum*** ** serovar 3 str. ATCC 700970**	Human	611	751719	AF222894	[45]
***U. parvum*** ** serovar 3 str. ATCC 27815**	Human	609	751679	CP000942	-
***M. synoviae*** ** 53**	Bird	659	799476	NC_007294	[46]
***M. pulmonis*** ** UAB CTIP**	Rodent	-	963879	AL445566	[47]
***M. pneumoniae*** ** M129**	Human	688	816394	U00089	[48]
***M. penetrans*** ** HF-2**	Human	1037	1358633	BA000026	[49]
***M. mycoides*** ** subsp. mycoides SC str. PG1**	Ruminant	1016	1211703	BX293980	[50]
***M. mobile*** ** 163K**	Fish	635	777079	AE017308	[51]
***M. hyorhinis*** ** HUB-1**	Swine	658	839615	NC_014448	[52]
***M. hyopneumoniae*** ** J**	Swine	657	897405	NC_007295	[46]
***M. hyopneumoniae*** ** 7448**	Swine	657	920079	NC_007332	[46]
***M. hyopneumoniae*** ** 232**	Swine	691	892758	NC_006360	[53]
***M. genitalium*** ** G37**	Human	475	580076	NC_000908	[5]
***M. gallisepticum*** ** str. R(low)**	Bird	763	1012800	AE015450	[54]
***M. crocodyli*** ** MP145**	Crocodile	689	934379	CP001991	-
***M. conjunctivae*** ** HRC**	Sheep and goats	696	846214	FM864216	[55]
***M. capricolum*** ** subsp. ATCC 27343**	Ruminant	812	1010023	CP000123	-
***M. arthritidis*** ** 158L3-1**	Rats and mice	631	820453	NC_011025	[56]
***M. agalactiae*** ** PG2**	Sheep and goats	759	877438	CU179680	-

**Figure 2:**
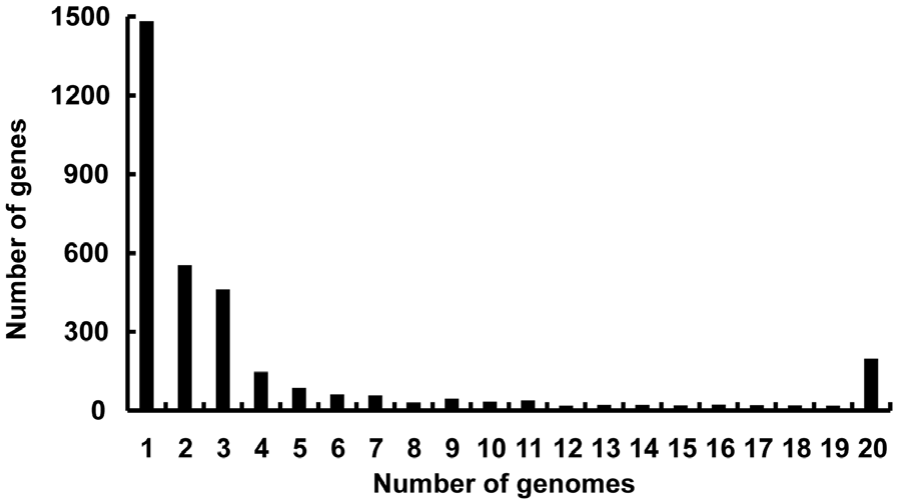
Frequency of genes within the 20 genomes included in this analysis. Genes present in a single genome represent lineage specific genes, while at the opposite end of the scale, genes found in all 20 genomes represent the *Mycoplasma* core genome.

**Figure 3:**
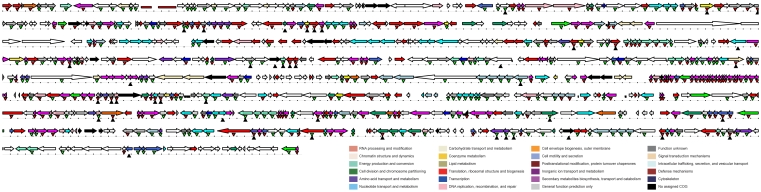
Viable core and pan genes distribution in the *M. hyorhinis* HUB-1 map. The *M. hyorhinis* HUB-1 genome is shown at a scale of 100 kb per line. Colored arrows above the line indicate annotated genes. Genes are colored according to their functional category, as indicated in the key. rRNAs and tRNAs are denoted by red and purple rectangles, respectively. Red triangles above the line represent the core genes of *Mycoplasmataceae*. Blue triangles above the line represent the genes shared by all the species in clade III, but which are absent from other lineages. Black triangles below the line represent the disrupted genes documented in *M. genitalium* G37, mapped onto the *M. hyorhinis* HUB-1 genome [5].

The extent of the pan-genome is opposed to the core. Genes that are in common between the different species within the family *Mycoplasmataceae* comprised our core genome - the set of orthologous genes determined the common properties of this family. In this work, the tribeMCL program was used to cluster orthologous genes, and a total of 13,654 predicted proteins were grouped into 3,355 clusters, each cluster representing a group of putative orthologs. The 1,481 genes that are present in single genomes (Figure 2) represent lineage specific genes. In addition, the 196 genes shared by all the 20 strains comprised our core genome (Figure 3).

### Functional Characterization of the Core Genome

The use of the core genome concept has led to important insights into the evolution of bacterial species and identification of potentially important novel genes [13]. In terms of functional assignments according to COGs, almost half (42.3%) of the proteins observed from the core genome are devoted to translation, ribosomal structure, and biogenesis (Figure S1). Our results support the analysis of Ouzounis and Kyrpides [14], who demonstrated that genetic processes such as translation are conserved and close to the original form. Strikingly, 10.6% of the observed core genes have resisted functional assignments according to COGs classification (Table S2), which highlights the need for better functional characterization of these genes. Furthermore, by comparing functional categories of the core genome with the categories of the genome of *M. hyorhinis* HUB-1, we noticed that a large array of proteins devoted to amino acid, carbohydrate transport and metabolism, as well as defense mechanisms, were sharply reduced. Our results support the analysis of Fraser *et al.* [9] and Himmelreich *et al.* [15], who demonstrated that both *M. genitalium* and *M. pneumoniae* lost all the genes involved in amino acid synthesis, and their survival is totally dependent on an exogenous supply of the complete spectrum of amino acids. Beyond this, the pronounced reduction of those functional categories observed in the core genome might be the further genetic evidence for gene loss in *M. genitalium* and *M. pneumonia* [9], [15].

### Persistent Nonessential Genes *vs.* Essential Genes

Identification of the core genome has important implications for a broad range of microbiological applications, such as determining the essentiality of genes derived from the core genome and deriving traits that correspond to a common ancestor (orthology) [4]. In this work, we classified the core genome into two classes according to persistence and essentiality: persistent nonessential genes and conserved essential genes (Figure 3). Glass *et al.* [5] performed a global transposon mutagenesis study and identified 100 putatively nonessential genes in *M. genitalium*. We mapped those nonessential genes onto the *M. hyorhinis* HUB-1 genome and 24 of them were persistent among *Mycoplasma* genomes.

Focusing on gene persistence, the essentiality of a gene is relative to a set of experimental conditions. It is quite different for a cell to survive in a laboratory setting, with plenty of supplied metabolites, compared to thriving in the wild, where it competes with other organisms for limited resources. Starvation or stresses are omnipresent, and the fitness effect of persistent genes may be essential for survival under transition from one environmental condition to another [16]. After transposon mutagenesis, disrupted genes may not be essential for growth in rich media, but their loss may lead to such a low fitness that its deletion will never be fixed in natural populations [16]. For example, Glass *et al.* [5] isolated six mutants involved in recombination and DNA repair: *recA*, *recU*, Holliday junction DNA helicases *uvrA* and *uvrB*, formamidopyrimidine-DNA glycosylase *mutM*, which excises oxidized urines from DNA, and a likely DNA damage inducible protein gene. Interestingly, we noticed that these six disrupted genes occur in the core genome set, which suggests that these disrupted persistent genes might be critical for growth in variable environments over long periods. The survival of these mutants is probably due to their tolerances of IS element within a relatively short period. According to Glass *et al.*, these six mutants grew more poorly after repeated passage, which indicates that those genes are also critical for bacterial survival [5]. Our results in this regard generally agree with the analysis by Glass *et al*. This analysis prompted us to explore gene essentiality by combining both the experimental approaches and comparative genomics analysis.

### Genome-Based Reconstruction of *Mycoplasmataceae* Phylogeny

**Figure 4:**
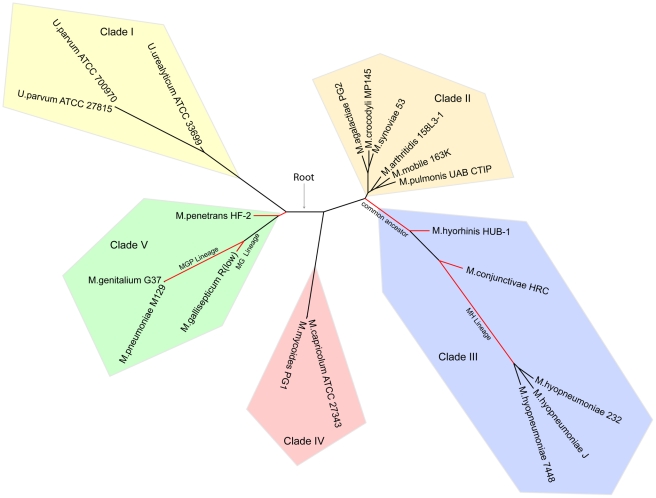
Phylogenetic tree of *Mycoplasmataceae*. The phylogenetic relationship was estimated and tested in one thousand bootstrap samples using TREE-PUZZLE version 5.2 with a BIONJ model (see Materials and Methods). This supertree shows five major distinct clades. The four lineages that were used as foreground in the branch-site model positive selection test are highlighted in red.

We constructed robust phylogenies for the family *Mycoplasmataceae* based on whole genome analysis. The supertree contains five major, distinct clades (Figure 4). In clade I, three *Ureaplasma* strains with a common host are clustered on a single branch. In clade II, six mycoplasma species (*M. pulmonis* etc.) with various lifestyles formed the cluster. In clade III, the strain HRC appears adjacent to strain HUB-1 and three isolates of *M. hyopneumoniae* strains are clustered closely, indicative of a recent common ancestry. In clade IV, *M. capricolum* and *M. mycoides* are clustered closely on a single branch and both of them are the agents of ruminant mycoplasmosis. In clade V, *M. genitalium* and *M. pneumoniae* are closely related, together with *M. penetrans* and *M. gallisepticum*, forming the cluster.

**Figure 5:**
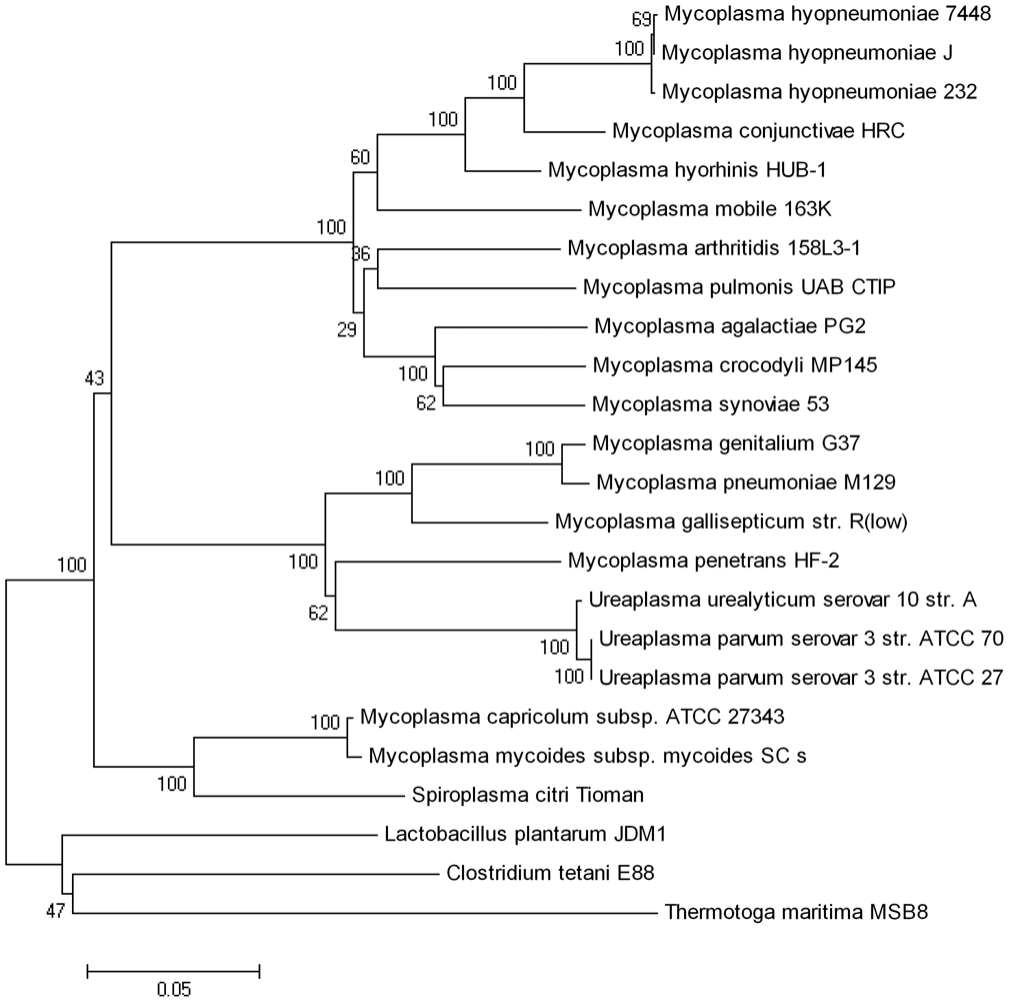
16S rRNA tree of *Mycoplasmataceae*. This consensus tree of 100 bootstrap replications was constructed based on 16S rRNA sequences using the Neighbor Joining (NJ) method implemented in MEGA 4.1. The bootstrap values are marked at the root of each branch.

Although single gene trees have been used extensively to estimate the species tree, evidence has shown that single gene trees may have particular difficulty in representing prokaryotic species phylogeny, because lateral gene transfer (LGT) occurs among prokaryotic genomes, and LGT may obscure the phylogenetic signal [17]. In this work, the 16S rRNA-based phylogeny has also been reconstructed for the family *Mycoplasmataceae* (Figure 5), which shows almost the same topology as the supertree. However, these two trees differ in the placement of *M. penetrans* HF-2 and *M. mobile* 163.

A recent study proposed that the “Tree of Life” may be resolved by concatenation of 31 orthologs occurring in 191 species [18], and an analogous approach has been applied to infer *Mycoplasmas* phylogeny by random concatenation of 91 protein sequences shared by 16 *mollicutes* [19]. The placement of *M. penetrans* in the concatenation-based phylogeny is consistent with the supertree (Figure 4).

### Positive Selection Analysis

We employed the branch-site test of Yang and Nielsen [20], [21] implemented in the program HY-PHY (http://www.datam0nk3y.org/hyphy/doku.php) to assess positive selection [22], [23] at particular sites in particular lineages. This method compares synonymous and nonsynonymous substitution rates in protein coding genes and regards a nonsynonymous rate elevated above the synonymous rate as evidence for positive selection. For the *Mycoplasma* data set, both swine-infecting and human-infecting lineages were tested. The branches we tested are highlighted in red on Figure 4.

In the four swine-infected lineages (HUB-1 lineage, MC lineage, MH lineage, and the common ancestor of clade III), 661 orthologous groups shared by all species in clade III were tested. A total of 23 genes were identified to be under positive selection (Table 2). These genes were assigned to functional categories according to the COG database. We found that a large fraction of the genes subject to positive selection were connected to DNA replication, recombination, and repair. Successful genome replication is essential for growth and survival of an organism, and polymerase complexes often fail to complete this task [24]. Also, replication is thought to contribute to proliferation and efficiency of the colonization of hostile environments [25]. Interestingly, we detected that positive selection occurs in the both of the *dnaA* and *dnaN* genes, which comprise the *oriC* region in *M. hyorhinis*, and in several copies of proteins connected to replication, recombination and repair in both MC lineage and the common ancestor of clade III. Selection pressure on these genes could reflect constraints on efficient genome replication during colonization and proliferation in the hostile environment of the host [25].

**Table 2:** Genes under Positive Selection in Swine-infecting Lineages.

Lineage	Gene	dN/dS^a^	Sequence %	COG(s)	Product
**MH Lineage**	mhp623	543.871	16.60%	COG1744R	ABC transporter
	mhp388	306.029	5.00%	COG3037S	ascorbate-specific PTS system enzyme IIC
	mhp603	4.42	0.00%	COG0195K	transcription elongation factor NusA
	mhp368	4.773	9.70%	COG0531E	putative membrane lipoprotein
	mhp595	515.577	13.40%	COG0266L	formamidopyrimidine-DNA glycosylase
	mhp480	1913.82	15.20%	-	hypothetical protein
**MC Lineage**	MCJ_005740	525.955	9.30%	COG1196D	putative ABC transporter ATP-binding protein P
	MCJ_003040	547.097	7.90%	COG0013J	alanyl-tRNA synthetase
	MCJ_007160	227.357	8.10%	COG2176L	DNA polymerase III PolC
	MCJ_002410	488.408	7.20%	COG4608E	oligopeptide ABC transporter ATP-binding protein
	MCJ_000340	316.719	5.10%	COG0556L	excinuclease ABC subunit B
**HUB-1 Lineage**	MHR_0001	2.122	7.10%	COG0593L	Chromosomal replication initiator protein dnaA
	MHR_0002	786.654	25.60%	COG0592L	DNA polymerase III beta subunit
	MHR_0009	8.155	5.50%	COG0525J	Valyl tRNA synthetase
	MHR_0132	306.624	7.30%	COG0060J	Isoleucyl tRNA synthetase
	MHR_0148	732.204	14.30%	COG0006E	Xaa-pro aminopeptidase
	MHR_0248	2.637	3.60%	COG0187L	DNA gyrase subunit B
	MHR_0318	180.115	16.40%	-	ABC transporter permease protein
	MHR_0377	6.345	9.70%	COG0202K	DNA-directed RNA polymerase subunit alpha
	MHR_0443	33.397	7.70%	COG0178L	Excinuclease ATPase subunit-like protein
	MHR_0609	14.416	16.70%	COG0544O	Trigger factor
**Common Ancester** b	MHR_0128	133.038	23.40%	COG0322L	Excinuclease ABC subunit C
	MHR_0131	33.679	13.50%	COG0188L	Topoisomerase IV subunit A
	MHR_0310	754.512	15.80%	COG2274V	ABC transporter ATP-binding and permease protein
	MHR_0486	58.164	12.90%	COG0587L	DNA polymerase III alpha subunit
	MHR_0489	3.669	29.10%	COG0532J	Translation initiation factor IF-2
	MHR_0639	8298.46	31.60%	-	Lipoprotein
	MHR_0363	6343.51	14.00%	COG1164E	Oligoendopeptidase F
	MHR_0356	6343.51	14.00%	COG1164E	Oligoendopeptidase F

a Ratio of the nonsynonymous to the synonomous mutation rate (dN/dS) measures the strength of selection, where values >1 indicate positive selection, and larger values indicate stronger selection.

b In the common ancestor lineage: a single gene of *M. hyorhinis* HUB-1 was used to represent each ortholog group (Table S1). Genes of *M. conjunctivae* HRC and three *M. hyopneumoniae* strains in the same ortholog group are also under positive selection.

In the case of human-infecting lineages, the lineage that stood out from the rest with regard to host specificity was *M. gallisepticum*, which is significantly associated with chronic respiratory disease in chickens [26]. Not surprisingly, this lineage was identified to be under the strongest selection pressure in clade V (Table S3). However, we failed to notice any selection pressure on the *dnaA* and *dnaN* genes in the MG lineage. A large number of genes related to DNA repair, RNA processing, Amino acid transport and metabolism were found under positive selection. Selection pressure on these genes may facilitate evolutionary flexibility in the MG lineage, hence its ability to adapt to new environments.

## Discussion

**Figure 6:**
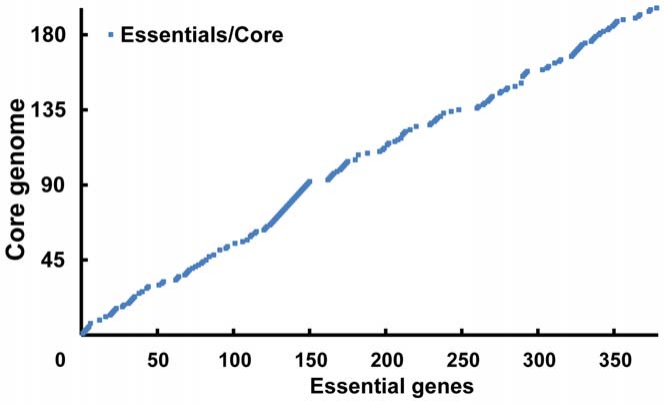
Similarity relationship between core and essential genes. Genes on x-axis represent the essential genes documented in *M. genitalium* G37, while genes on y-axis represent the core genome identified in this study. Both the genes on x-axis and y-axis are distributed in terms of genomic coordinates. On-diagonal, genes that are both essentials and core are indicated as a series of colored dots.

Numerous global transposon mutagenesis studies of minimal genomes have been performed to identify essential genes [5], [27]. Long-term natural selection can also delete nonessential genes in a wide range of species [6], [7], which is similar to, but more robust than, manual mutagenesis studied. With a minimal metabolism and little genomic redundancy, mycoplasmas are regarded as optimal microbes for the identification of essential genes [1]. It is believed that the *Mycoplasmas* evolved from a more conventional progenitor in the *Firmicutes* taxon by a process of massive genome reduction [16]. We found that each of the species in *Mycoplasmataceae* has undergone a similar process. These species have undergone various natural selection pressures in different environments. Most of the core genes remaining for such a long time should be considered as the essential genes needed by all the species within this family. Generally speaking, our comparative analysis was highly consistent with the studies by Glass *et al.* (Figure 6); however, we identified more genes that may have been deleted due to natural selection. The six genes differing between the two studies are all key genes of DNA replication and DNA repair: *recA*, *recU*, Holliday junction DNA helicases *uvrA* and *uvrB*, *mutM* and a likely DNA damage inducible protein gene. Those genes are core genes, but were disrupted in transposon mutagenesis studies [5]. Interestingly, Glass *et al.* stated that these six mutants grew more poorly after repeated passage, probably due to an accumulation of cell damage over time. This indicates that these six disrupted core genes may be critical for bacterial survival, especially over long periods. Therefore, we classified these genes as truly essential genes.

Although transposon mutagenesis has proven to be a useful method and has been used extensively to determine the essentiality of genes [27], this method is highly dependent on environmental conditions. For the most part, mutagenesis studies performed under nutrient-rich conditions provide a substantial underestimate of the number of genes that are essential under host environmental conditions [28]. In reality, it is quite different for a cell to survive in a laboratory setting and to thrive in the wild. Transposon mutagenesis might misclassify nonessential genes that slow growth without arresting it but can also miss essential genes that tolerate transposon insertions [4]. However, comparative genomics analysis has also limitations, since it is likely to underestimate the core genome because it takes into account only the genes that have remained similar enough during the course of evolution to be recognized as true orthologues. Therefore, it will not include genes with a high rate of evolution, which may not show their relationship in comparisons of distant taxons [4]. Taken together, both the experimental approaches and comparative genomics analysis should be taken into account when addressing questions of essentiality. Besides, the core genes set proposed in the current study are only essential for most species within this family. We failed to estimate the conserved genes needed for single species. Each member of a particular species was maintained in a distinct ecological niche, in which some of the genes that were not present in core genome may also be important for the mycoplasma survival. These genes may be termed “lineage specific” essential genes. As more genome sequence becomes available in the future, there will be an opportunity to explore more properties of species special core genes set using comparative genomic tools.

To date, most mycoplasma phylogenies have been derived from single gene comparisons, or from concatenated alignments of a small number of genes. The increasing availability of mycoplasma genomes presents an opportunity to reconstruct evolutionary events using entire genomes [29], [30]. As a tool for future comparative phylogenetic studies, we used both supertrees and single gene alignments to infer relationships between 20 strains of the family *Mycoplasmataceae*. Our supertree and 16S rRNA phylogenies are consistent in most of their branches. However, there are conflicts regarding whether *M. penetrans* is clustered with the *Ureaplasma* lineage or with Clade V, as well as the placement of *M. mobile* 163. We also compared our trees with a recent study, in which the phylogeny of the *Mycoplasmas* was reconstructed by random concatenation of 91 protein sequences shared by 16 *mollicutes* [19]. The placement of *M. penetrans* in the concatenation-based phylogeny is consistent with the supertree, while the bootstrap value ( = 62) of the branch of *M. penetrans* and *Ureaplasma* in the 16S rRNA tree is low. Therefore, we placed *M. penetrans* into clade V. The location of *M. mobile* 163 is different among all three trees, which indicates a complicated phylogenic history for this strain, which may have involved recombination or other LGT events.

Phylogenetic reconstruction based upon concatenation of multiple orthologous genes can generate a more accurate tree than that done with a single gene [31], [32]. The supertree is even better than the concatenation-based tree, because it is immune to long-branch attraction artifacts [33], [34]. Thus, a robust supertree was constructed in this study to present the phylogeny of the family *Mycoplasmataceae*. The supertree was then used as a foreground for further analysis. Based on the supertree of *Mycoplasmataceae*, we classified these twenty strains into five different clades, between which the host specificity varies. All three strains in Clade I and three of the four strains in Clade V (except *M. gallisepticum*) were identified to be agents of human infection; therefore, these two clades form the human-infecting lineage. Both of the two sequenced strains in clade IV are the agent of ruminant infection, and thus represent the ruminant-infecting lineage. Four of the five strains in Clade III (except *M. conjunctivae*) are associated with swine mycoplasmosis and form the swine-infecting lineage. Briefly, most species with the same host specificity clustered together, forming a separate clade. There appears to be an approximate correlation between the divergence of species and the level of positive selection detected in different lineages. We suspect that host specificity was determined after the emergence of *Mycoplasma* species. Subsequent host jumping events may have been caused by a series of natural selection events during evolution.

To gain deeper insights into the molecular evolution events underlying natural selection, we employed the branch-site method to assess positive selection in swine-infecting and human-infect lineages. According to Petersen *et al.* [35], two categories of genes, immune-related and environmental adaptation related genes, are expected to show strong evidence for positive selection. Our analysis revealed that a number of genes related to DNA replication and repair (*dnaA*, *dnaN*, *gyrB*, *uvrA*, *polC*, *uvrB*, *uvrC*, *parC*, *dnaE*), show remarkably strong evidence for positive selection. These genes were unevenly distributed across HUB-1, the MC lineage and the common ancestor of clade III. Notably, both the *dnaA* and *dnaN* genes, which compose the *oriC* region, were identified to be under positive selection in the HUB-1 lineage. Previous studies have already demonstrated that replication may contribute to proliferation and efficiency of the colonization of hostile environments [25]. Therefore, we suspected that selection pressure on *oriC* may be one of the reasons why *M. hyorhinis* can thrive among different species of cell lines.

This research provides a better insight into, and understanding of, persistent nonessential genes, and encourages exploration of essential genes by combining both the experimental approaches and comparative genomics analysis. This study also provides a comparative genomics method for addressing questions of essentiality. With the increasing number of genome sequences available for the same species in the future, this method will be useful for exploring species-specific essential genes.

## Materials and Methods

### Bacterial Strains and Genome Sequences

*M. hyorhinis* strain HUB-1 was isolated from the respiratory tract of swine in China and confirmed to be an *M. hyorhinis* strain by verifying the 16S rRNA region. The main characteristics of 20 *Mycoplasmas* strains with freely available genomes at the time of the study are presented in Table 1. These genomes were used for comparative analysis.

### Assignment of Orthologs and Phylogenetic Analysis

We analyzed *M. hyorhinis* HUB-1 and 19 other *Mycoplasmataceae* genomes from the NCBI databases. To ensure consistency, the annotations of all genomes were verified based on the similarity with *M. hyorhinis* HUB-1, using the tBLASTn algorithm [36]. The sets of orthologous protein-coding genes were defined as mutual fully transitive reciprocal BLASTP [37] hits (with E-value<10^−4^) [38]. Co-ortholog groups were identified by the method similar to Inparanoid [39] and ortholog gene clusters were obtained using the tribeMCL program [40]. The nucleic acid sequence of each ortholog group was aligned using the CLUSTALW program version 1.83 [41]. For each data set, the phylogenetic relationship was estimated and tested in one thousand bootstrap samples using TREE-PUZZLE version 5.2 (general time reversible (GTR) +Γ4+I model of evolution with a BIONJ starting tree) [42]. The bi-partitions with at least 70% support from the bootstrap test in each data set were recorded as “0/1” status and used to reconstruct the consensus sequence. The phylogenetic relationship of the consensus sequence was built using the SplitsTREE 4 with the BioNJ model.

### Positive selection analysis

We employed the branch-site test of Yang and Nielsen [20], implemented in the program HY-PHY, to assess positive selection at particular sites and lineages. Briefly, the likelihood of a model that does not allow positive selection is compared to one allowing positive selection on some specified lineages. The model allowing positive selection is tested using a likelihood ratio test (LRT) [43] that is compared to a χ2 statistic with two degrees of freedom. Likelihoods were estimated on the genes or species trees. For the *Mycoplasma* data set, both swine-infecting and human-infecting lineages were tested (Figure 4). To avoid the interference of recombination, only genes that support all four lineages in their gene trees (with >70% bootstrap support) were used. In total, 661 genes were tested. Finally, p values were corrected for multiple hypotheses testing using the Bonferroni method [44].

## Supporting Information

Figure S1 Comparison of COG Distribution in the Core Genome and in *M. hyorhinis* HUB-1.
(TIF)

Table S1 Gene Content Table: Composition of Each Gene Cluster per Genome.
(XLS)

Table S2 Characteristics of the Core Genome Identified in the Family *Mycoplasmataceae*.
(XLS)

Table S3 Genes under Positive Selection in the Human-infecting Lineage.
(XLS)
