# Supplementary figures and images for: Comparative Genomics of Mycoplasma: Analysis of Conserved Essential Genes and Diversity of the Pan-Genome
Source: PLoS One. 2012 Apr 20;7(4):e35698. doi: 10.1371/journal.pone.0035698 (PMC3335003; doi:10.1371/journal.pone.0035698)

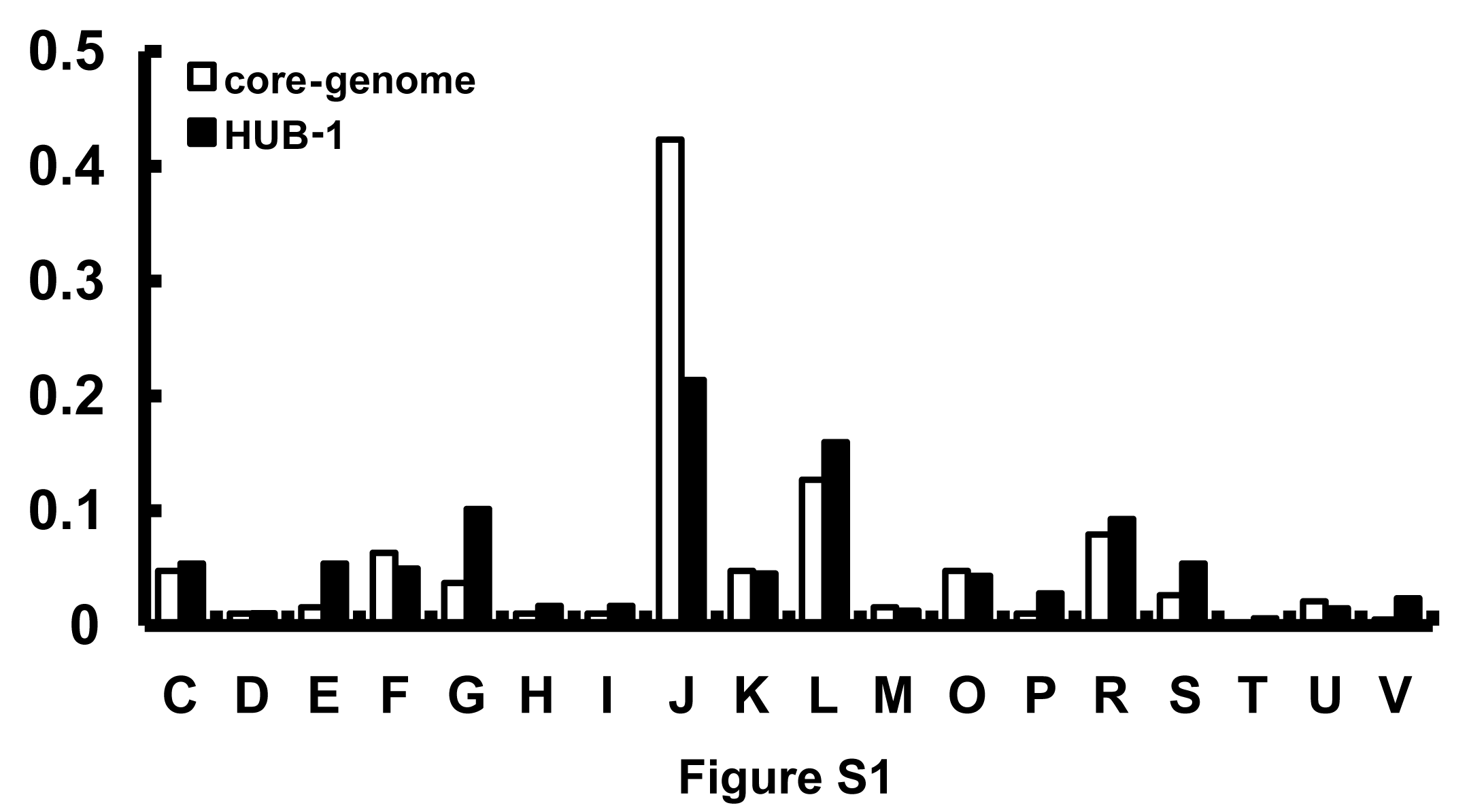

Supplement: Figure S1 — Comparison of COG Distribution in the Core Genome and in M. hyorhinis HUB-1. (TIF) [file pone.0035698.s001.tif]
